# Health Professionals’ eHealth Literacy and System Experience Before and 3 Months After the Implementation of an Electronic Health Record System: Longitudinal Study

**DOI:** 10.2196/29780

**Published:** 2022-04-29

**Authors:** Lars Kayser, Astrid Karnoe, Emily Duminski, Svend Jakobsen, Rikke Terp, Susanne Dansholm, Michael Roeder, Gustav From

**Affiliations:** 1 Section of Health Service Research Department of Public Health University of Copenhagen Copenhagen Denmark; 2 Department of Internal Medicine Herlev-Gentofte Hospital Hellerup Denmark

**Keywords:** health care professionals, eHealth literacy, electronic health record, implementation, digital health, eHealth, health literacy, health records, eHealth records, patient care

## Abstract

**Background:**

The implementation of an integrated electronic health record (EHR) system can potentially provide health care providers with support standardization of patient care, pathways, and workflows, as well as provide medical staff with decision support, easier access, and the same interface across features and subsystems. These potentials require an implementation process in which the expectations of the medical staff and the provider of the new system are aligned with respect to the medical staff’s knowledge and skills, as well as the interface and performance of the system. Awareness of the medical staff’s level of eHealth literacy may be a way of understanding and aligning these expectations and following the progression of the implementation process.

**Objective:**

The objective of this study was to investigate how a newly developed and modified instrument measuring the medical staff’s eHealth literacy (staff eHealth Literacy Questionnaire [eHLQ]) can be used to inform the system provider and the health care organization in the implementation process and evaluate whether the medical staff’s perceptions of the ease of use change and how this may be related to their level of eHealth literacy.

**Methods:**

A modified version of the eHLQ was distributed to the staff of a medical department in Denmark before and 3 months after the implementation of a new EHR system. The survey also included questions related to users’ perceived ease of use and their self-reported information technology skills.

**Results:**

The mean age of the 194 participants before implementation was 43.1 (SD 12.4) years, and for the 198 participants after implementation, it was 42.3 (SD 12.5) years. After the implementation, the only difference compared with the preimplementation data was a small decrease in staff eHLQ5 (*motivated to engage with digital services*; unpaired 2-tailed *t* test; *P*=.009; effect size 0.267), and the values of the scales relating to the medical staff’s knowledge and skills (eHLQ1-3) were approximately ≥3 both before and after implementation. The range of scores was narrower after implementation, indicating that some of those with the lowest ability benefited from the training and new experiences with the EHR. There was an association between perceived ease of use and the 3 tested staff eHLQ scales, both before and after implementation.

**Conclusions:**

The staff eHLQ may be a good candidate for monitoring the medical staff’s digital competence in and response to the implementation of new digital solutions. This may enable those responsible for the implementation to tailor efforts to the specific needs of segments of users and inform them if the process is not going according to plan with respect to the staff’s information technology–related knowledge and skills, trust in data security, motivation, and experience of a coherent system that suits their needs and supports the workflows and data availability.

## Introduction

### Background

During the past 50 years, technological and digital evolution has facilitated the transformation of the organization and delivery of health services [1]. For more than two decades, it has been anticipated that electronic health records (EHRs), also termed electronic medical records or electronic patient records, would provide more efficient, effective, and safe workflows, benefiting both providers and patients [2-8].

In many regions, primarily Europe and the United States, the first generations of EHR were launched in the late ’90s or the early 2000s [8,9]. These systems have been either upgraded or replaced with new systems with more advanced features and the integration of functions from various specialized systems into one system. The new generations of EHR have the potential to support standardization of patient care, pathways, and workflows, as well as provide organizations with data for business intelligence and health care professionals with decision support, easier access, and the same interface across its features and subsystems [8].

### Factors for Medical Staff’s Acceptance of an EHR

Not all implementations of EHR systems have been successful over the years. The reasons for this may be understood in relation to the context, content, and processes of EHR [9]. This includes the structure and digital maturity of the organizations, influence on or interaction with existing workflows, degree of involvement, digital experience, and competence of the staff [2,4,8-10].

The attitude of the medical staff toward a new EHR, as well as their capability to gain benefits, is related to the staff’s level of information technology (IT) or eHealth literacy (eHL) [4,8,10]. To accept and adopt the technology, the user needs to feel confident and expect a good user experience based on the perceived ease of use and usefulness [4].

In general, most studies on the adoption and acceptance of technology build on the Technology Acceptance Model (TAM) or the Unified Theory of Acceptance and Use of Technology (UTAUT) [4,11,12]. Both TAM and UTAUT are relatively old theories that have not been specifically developed for the health care sector but have been adapted in several studies in the context of health [4]; for example, performance expectancy (individuals believe that the use of technology will be beneficial), effort expectancy (expected ease of use), social influence (expected attitude of significant others toward using the technology), and facilitating conditions (organizational or technical resources and preconditions to technology use) [10].

In 2015, Monkman and Kushniruk [13] proposed the *Consumer Health Information System Adoption Model*. The model is based on a theoretical approach and suggests, in alignment with the TAM and UTAUT, that an essential factor for adoption is the user experience; more importantly, they proposed that user experience relates not only to usability and perceived ease of use or usefulness but also to the individual user’s level of eHL as the user’s level affects their perceived user experience and influences the requirements of the systems interface [13].

### eHealth Literacy

eHL was introduced to describe the competences needed to engage with digital health solutions in a health context. eHL was originally conceptualized by Norman and Skinner [14], who also proposed the first definition: “the ability to seek, find, understand, and appraise health information from electronic sources and apply the gained knowledge to addressing or solving a health problem” [14]. In 2015, Nørgaard et al [15] challenged the original concept with the proposal of a new and more comprehensive model, the eHL Framework (eHLF). The eHLF comprises 7 dimensions that not only address the user’s knowledge and skills, similar to Norman and Skinner [14], but also address the interface and context (ie, the user’s trust, motivation, and experience with digital services and technology).

The dimensions relating to the user’s knowledge and skills are eHLF1, *ability to process information*; eHLF2, *engagement in own health*; and eHLF3, *ability to actively engage with digital services*. The dimensions relating to the user’s trust in the way their health data are handled and the benefits of digital services are eHLF4, *feel safe and in control*, and eHLF5, *motivated to engage with digital services*. The final two dimensions, eHLF6, *access to digital services that work*, and eHLF7, *digital services that suit individual needs*, relate to the experience of the available digital services in relation to access to relevant information whenever it is needed in a way that suits the user’s needs [15]. The user’s self-reported capability within the 7 dimensions can be quantified using the eHL Questionnaire (eHLQ), which is based on the eHLF [16].

Both the eHLF [17,18] and the eHLQ may, alone [19,20] or in combination with other scales such as the Readiness and Enablement Index for Health Technology instrument [21-23], help identify potential barriers or facilitators with respect to the user’s capabilities, their trust and motivation, and their experiences with digital services.

In the context of the implementation of an EHR, assessment of eHL among medical staff has the potential to provide the supplier and health care organization before the implementation with insights into which groups may have particular needs to be addressed and after the implementation with insights into how the implementation, including educational programs, affects users’ knowledge, skills, motivation, and experience.

### The Setting of the Study

Part of the validation of the eHLQ was the inclusion of data from a medical outpatient clinic in the Capital Region of Denmark, Herlev-Gentofte University Hospital, from November 2015 to March 2016 [19]. Incidentally, one of the largest implementations of an EHR in northern Europe was planned to take place simultaneously at the same hospital.

The new integrated EHR system was planned to replace >20 existing systems and be followed by investment in new technologies such as handheld devices, mobile computers, and standardized equipment such as infusion pumps. At the time of implementation, the medical staff was used to using a traditional EHR supporting documentation in notes along with a laboratory system, an imaging system, and a medicine prescription system, as well as a patient administrative system primarily used by medical secretaries and a documentation system for nurses. The latter was used by medical physicians, nursing assistants, and registered nurses. Everyday use was supported by both local health professionals trained as superusers and by regional IT support with a help desk.

The Capital Region of Denmark’s expected outcomes of the introduction of the new integrated EHR were more efficient workflows that were better supported by technology and a reorientation of the professional roles and tasks, including easier and better communication with outpatients [24]. An important change in workflow was the introduction of the principle that the individual staff member responsible for an order should also enter this into the system, which changed the work balance among medical physicians, registered nurses, and medical secretaries [24]. The introduction of an anticipated, easier to use EHR better supporting communication and workflows, together with a 3 full days training program for the nursing assistants and ≥4 full days for the other groups of medical staff, followed by 2 weeks of intensive support by superusers and specially trained *floor walkers* after the launch of the new EHR system, led us to expect that the overall effect of the implementation would be an increase in the medical staff’s eHL and their perceived ease of use.

The combination of having a new instrument to assess the multifaceted dimensions of eHL during the implementation of a promising new suite of EHR components, as well as curiosity about how this would influence the medical staff’s eHL profile, led us to initiate this study. Our aim is to evaluate the eHL of the medical staff using the eHLQ before and 3 months after the implementation of the EHR to examine the overall effect of the introduction of the new system.

We worked from the hypotheses presented in Textbox 1.

**Hypotheses of this study.**

**Hypothesis 1**

- Hypothesis 1.1: The medical staff’s personal knowledge and skills (eHealth Literacy Questionnaire [eHLQ] 1-3) will increase as a consequence of the introduction of the new electronic health record (EHR) with a 3- to 4-day training program and extensive support for the first 2 weeks after implementation.
- Hypothesis 1.2: An overall positive experience with the new system with an EHR will improve the sense of feeling safe and in control (staff eHLQ4) and increase motivation (staff eHLQ5) as the medical staff experience the expected benefits of an integrated EHR system.
- Hypothesis 1.3: The implementation will provide an experience of an EHR that brings data together, makes them easier to access (staff eHLQ6), and better suits the individual needs (staff eHLQ7).
- Hypothesis 1.4: The eHLQ scores may differ between the groups of medical staff because of different professional cultures, tasks, obligations, and responsibilities.

**Hypothesis 2**

- Hypothesis 2.1: The experience of ease of access, ease of data sharing, and stability of the information technology system will increase with the new integrated system running on a more stable platform.
- Hypothesis 2.2: The increase may be associated with staff eHLQ5, staff eHLQ6, and staff eHLQ7, establishing a possible association between factors known to be important for technology acceptance and eHealth literacy dimensions.

To explore these hypotheses, we formulated the following research questions (RQs):

- RQ1: What is the level of the medical staff’s eHL before and 3 months after the implementation of the new EHR?
- RQ2: How do medical staff perceive ease of use, as measured by the ease of access, ease of data sharing, and stability of the existing EHR before implementation, compared with the new integrated EHR system after implementation, and are there any differences between professions?
- RQ3: Is there an association between the scores of staff eHLQ5-7 and perceived ease of use, as measured by ease of access, ease of data sharing, and stability of the system?

## Methods

### Overview

The study was originally designed as a longitudinal study to evaluate the medical staff’s eHL, perceived ease of use, and use of functions before implementation and at 3 and 12 months after implementation. The involved department was restructured before month 12 by fusing with 2 other medical departments, resulting in a change of jobs for 3 of the 4 clinical working authors of this study and relocation of the acute clinical unit and other specialties such as gastroenterology from one location in the city of Gentofte to another location in the city of Herlev. Therefore, we had to exclude the 12-month follow-up, as it was not feasible for us to conduct. A planned complementary qualitative study was also not feasible in the initial period because of a lack of support from a higher level of the organization responsible for the implementation.

The study was designed with 2 cross-sectional samples, inviting all the medical staff employed at 2 time points. In March 2016, an invitation was sent by email to the medical staff working in all units, including the outpatient clinic, at the Department of Medicine C, Herlev-Gentofte University Hospital, containing a link to the survey, and by mid-March, a reminder to participate was sent to those who did not initially respond. The second survey was sent out in September 2016. The study was endorsed by the head of the department, who took an active part in recruiting respondents at both time points.

The survey was sent to 295 medical staff members in both rounds, with a response rate of 65.8% (194/295) in the first round and 67.1% (198/295) in the second round and respondents answering some or all questions. The distribution of respondents among different groups of medical staff is presented in Table 1.

**Table 1:** Distribution of medical staff^a^.

Staff	Before implementation (N=194), n (%)	After implementation (N=198), n (%)
Medical physician	46 (23.7)	50 (25.3)
Medical secretary	29 (14.9)	26 (13.1)
Nursing assistant	16 (8.2)	15 (7.6)
Registered nurse	97 (50)	104 (52.5)
Other professions	6 (3.1)	3 (1.5)

^a^The table includes respondents who answered some or all questions.

All groups of medical staff employed at the department were represented in response to the survey. For this study, we report on all respondents in relation to overall statistics but have not included the group of other professions (9/295, 3.1%) when reporting on groups of professional medical staff (ie, medical physicians, medical secretaries, nursing assistants, and registered nurses). In Denmark, these 4 professional groups have the qualifications and level of education according to the International Standard Classification of Education (ISCED) given in the following sections [25].

Medical physicians had a master’s degree in medicine at ISCED level 7 [25]. Some of them also held a PhD or medical thesis degree at ISCED level 8. Their experience ranged from registrars leaving the medical school to consultants, who were specialists. Registered nurses had 3.5 years of education and held a bachelor’s degree in nursing at ISCED level 6. Nursing assistants had a vocational education, which currently is 3 years at ISCED level 4; however, some of the respondents may have had a previous education of 2 years at ISCED level 3. Medical secretaries also had a vocational education of 3 years, with specialization in the medical field.

The surveys in the project were intentionally designed so that they would not be misinterpreted as an evaluation of the new EHR. In accordance with the hypotheses and RQs, the sole intention of the surveys was to describe the change in the medical staff’s eHL and their perceived ease of use of the 2 different EHR solutions.

The survey comprised four sections: (1) digital experience; (2) the staff eHLQ; (3) experience of use with the EHR, including perceived ease of use; and (4) use of functions of the EHR. The use of functions and components will be reported elsewhere and are not included here.

Sex, age, and professional roles were extracted from the administrative system and merged with the survey. This was performed by an administrator based on each participant’s unique employee identifier. After the merging was complete, person-identifiable data were removed from the file, which was then handed over to the author group for analysis.

### Digital Skills

As an indicator of experience with digital services in their private lives, the respondents were asked to report on their use of the national digital mail service called e-Boks. e-Boks facilitates all communication from public authorities in Denmark to citizens aged >15 years. Individuals with language difficulties or disabilities can be exempted from the e-Boks system. The respondents reported on their use with four response options—rarely or never, at least once every 6 months, at least once a month, and at least once a week—scored from 1 to 4.

The second question was how their colleagues would describe their user level in relation to the systems they used at work with three options—standard user, advanced user, or expert user—scored from 1 to 3.

### Staff eHLQ

The staff eHLQ is a modified version of the eHLQ [16]. The modification comprised rephrasing 12 items in scales 4 to 7 of the eHLQ to change the perspective of the respondent from themselves to their interaction with patients; for example, item 24, which was changed from “I find I get better services from my healthcare provider when I use...” to “I find that patients receive better services from health professionals when...” The items in staff eHLQ1-3 are equivalent to the validated eHLQ, except that 1 item in eHLQ1 is missing because the staff eHLQ used here was based on an earlier version of the eHLQ.

Therefore, the staff eHLQ in this study comprised 34 items covering seven dimensions of eHL in the following scales: eHLQ1*, using technology to process health information*; eHLQ2, *understanding of health concepts and language*; eHLQ3, *ability to actively engage with digital services*; staff eHLQ4, *feeling safe and in control*; staff eHLQ5, *motivated to engage with digital services*; staff eHLQ6, *access to digital services that work*, and staff eHLQ7, *digital services that suit individual needs*. The eHLQ1 and staff eHLQ7 scales comprise 4 items, eHLQ2 to staff eHLQ5 comprise 5 items, and staff eHLQ6 comprises 6 items. The response options ranged from strongly disagree to strongly agree and were scored from 1 to 4 [16].

The validation of the eHLQ was reported by Kayser et al [16]. To ensure that the aforementioned minor changes did not change the internal consistency, we calculated Cronbach α, which is similar to those initially reported with the following values: eHLQ1=.7519, eHLQ2=.7646, eHLQ3=.8413, eHLQ4=.7463, eHLQ5=.7422, eHLQ6=.6786, and eHLQ7=.8131.

### Perceived Ease of Use Evaluated as the Experience of Use With the Digital Information and IT Systems

This part comprised three items adopted from a national, regular survey, *Termostaten*, administrated by *The Danish e-Observatory* [26], which assesses users’ self-reported experience of the following three items:

1. Quick and easy access: “In my daily work I have quick and easy access to all the essential digital information from my own sector or unit (department or hospital) that I need.”
2. Sharing of data to reduce doublet registration: “In my daily work I experience, that data is shared between systems in a way that makes double registrations avoidable.”
3. Stability of systems: “In my daily work I experience, that the work-related IT-system I use every day are stable and function without operational problems or crashes.”

The 3 items are all considered to report on perceived ease of use and are used for this purpose in the analysis. The response options ranged from strongly disagree to strongly agree, with scores ranging from 1 to 4. Each of the 3 items was evaluated separately.

### Statistical Analysis

We treated the 2 samples as independent in the analysis as the questionnaire was administered anonymously to us.

To test hypotheses 1.1, 1.2, 1.3, and 2.1, we used an unpaired 2-tailed *t* test to compare the levels of eHL and perceived ease of use before and after the implementation. The effect size was calculated as Cohen *d*, and 1-way ANOVA was used to examine significant differences between the 4 medical staff groups in terms of their scores. The Tukey honest significant difference test was used a posteriori to determine which medical staff groups’ means differed significantly from each other. We also used an unpaired *t* test to examine differences in scores between males and females. Pearson *r* was used to examine the association between age in relation to the eHLQ scales and self-reported IT skills.

To test hypothesis 2.2, Pearson *r* was calculated to examine the correlations among the experience of quick and easy access; sharing of data to reduce doublet registration; and the stability of the IT system; and staff eHLQ5, staff eHLQ6, and staff eHLQ7.

All quantitative data are reported as means and SDs.

Statistical calculations were performed using Stata (version 16; StataCorp).

### Ethics Approval

Under Danish law, permission from an ethics committee was not required as biological material was not obtained or processed in the study, and no clinical intervention of the respondents was performed. The data were gathered by the hospital administration and stored on their servers. The anonymized data were further processed at the University of Copenhagen. Before data collection, all respondents were introduced to the survey by their local leaders. When initiating the survey, the respondents provided informed consent to participate by filling in the survey.

## Results

### Overview

The age and sex distributions of the 2 samples are presented in Table 2. The mean age of the sample before implementation was 43.1 (SD 12.4) years and 42.3 (SD 12.5) years in the sample after implementation. The sample mainly comprised female respondents.

Most respondents used the national email system, e-Boks, regularly. Few medical physicians and nursing assistants did not use the national email service before the implementation of the EHR. After implementation, all groups used the service at least once every 6 months, and most of them used the service more regularly. The average scores were approximately the same before and after implementation (Table 3). The score of how a colleague described their IT skills did not change over time and did not differ between the medical staff groups. Before implementation, there was a minor negative correlation with age (*r*=–0.1965; *P*=.009), which increased 3 months after implementation (*r*=–0.283; *P*<.001), signifying that the younger members of staff were more confident in their IT skills, a difference that increased after the introduction of the new EHR system. We also found a difference in males scoring higher than females both before implementation (mean 2.074, SD 0.675 vs mean 1.516, SD 0.661; *P*<.001; effect size 0.83) and 3 months after implementation (mean 1.848, SD 0.712 vs mean 1.538, SD 0.627; *P*=.01; effect size 0.482).

**Table 2:** Background variables by job functions.

Characteristics and staff			Before implementation (N=194)	After implementation (N=198)
**Age (years), mean (SD; range)**				
	Overall		43.1 (12.4; 23-68)	42.3 (12.5; 24-68)
	Medical physician		43.6 (12.7; 26-68)	42.0 (13.0; 27-68)
	Medical secretary		49.3 (10.7; 24-64)	50.1 (9.8; 25-63)
	Nursing assistant		52.4 (8.7; 34-64)	54.8 (9.0; 35-66)
	Registered nurse		39.3 (12.0; 23-66)	38.8 (11.3; 30-60)
**Sex, n (%)**				
	**Overall**			
		Male	29 (14.9)	35 (17.7)
		Female	165 (85.1)	163 (82.3)
	**Medical physician**			
		Male	16 (8.2)	22 (11.1)
		Female	30 (15.5)	28 (14.1)
	**Medical secretary**			
		Male	2 (1)	0 (0)
		Female	27 (13.9)	26 (13.1)
	**Nursing assistant**			
		Male	1 (0.5)	2 (1)
		Female	15 (7.7)	13 (6.6)
	**Registered nurse**			
		Male	10 (5.2)	10 (5.1)
		Female	87 (44.8)	94 (47.5)

**Table 3:** Information technology skills by job functions.

Skill and staff		Before implementation, mean (SD; range)	After implementation, mean (SD; range)
**Use of e-Boks**		3.301 (0.615; 1-4)	3.354 (0.558; 2.0-4.0)
	Medical physician	3.217 (0.629; 2-4)	3.3 (0.544; 2.0-4.0)
	Medical secretary	3.310 (0.660; 1-4)	3.35 (0.485; 3.0-4.0)
	Nursing assistant	3.375 (0.806; 1-4)	3.67 (0.488; 3.0-4.0)
	Registered nurse	3.333 (0.556; 2-4)	3.346 (0.587; 2.0-4.0)
**Information technology** **skills described by colleague**		1.601 (0.692; 1.0-3.0)	1.594 (0.65; 1.0-3.0)
	Medical physician	1.667 (0.6396; 1.0-3.0)	1.553 (0.619; 1.0-3.0)
	Medical secretary	1.963 (0.854; 1.0-3.0)	1.783 (0.736; 1.0-3.0)
	Nursing assistant	1.2 (0.414; 1.0-2.0)	1.286 (0.726; 1.0-3.0)
	Registered nurse	1.547 (0.663; 1.0-3.0)	1.61 (0.618; 1.0-3.0)

### eHLQ Scales

After 3 months from the implementation, the only difference compared with the preimplementation data was a decrease in staff eHLQ5 (*motivated to engage with digital services*; unpaired *t* test; *P*=.009; effect size 0.267), whereas the other scales did not differ from before implementation (effect size ranging from 0.0093 to 0.0916). As seen in Table 4, the eHLQ scores in relation to the respondents’ knowledge and skills (eHLQ1-3) were approximately ≥3 both before and after implementation. The range of scores was narrower after the implementation, indicating that some of those with the lowest ability benefited from training and new experiences with the EHR. On the basis of these findings, we rejected hypotheses 1.1 to 1.3. With respect to hypothesis 1.4, we found differences among the groups of medical staff for some of the scales, both before and after the implementation of the EHR, which partly supports our hypothesis.

**Table 4:** eHLQ^a^ scores by professional groups.

Scales and staff			Before implementation, mean (SD; range)		After implementation, mean (SD; range)
**eHLQ1: using technology to process health information**			2.980 (0.597; 1.0-4.0)		3.009 (0.574; 1.5-4.0)
	Medical physician	2.989 (0.570; 1.75-4.0)		2.893 (0.663; 1.5-4.0)	
	Medical secretary	2.896 (0.611; 1.0-4.0)		2.860 (0.479; 2.0-4.0)	
	Nursing assistant	2.921 (0.778; 1.0-4.0)		3.233 (0.458; 2.3-4.0)	
	Registered nurse	3.018 (0.565; 1.5-4.0)		3.046 (0.544; 2.0-4.0)	
**eHLQ2: understanding of health concepts and language**			3.399 (0.467; 1.0-4.0)		3.407 (0.439; 2.0-4.0)
	Medical physician	3.565 (0.356; 2.6-4.0)		3.551 (0.429; 2.0-4.0)	
	Medical secretary	3.255 (0.487; 2.4-4.0)		3.160 (0.374; 2.4-3.8)	
	Nursing assistant	3.163 (0.742; 1.0-4.0)		3.413 (0.389; 3.0-.4.0)	
	Registered nurse	3.408 (0.426; 2.4-4.0)		3.389 (0.441; 2.0-4.0)	
**eHLQ3: ability to actively engage with digital services**			3.359 (0.505; 1.0-4.0)		3.364 (0.502; 1.8-4.0)
	Medical physician	3.448 (0.458; 2.4-4.0)		3.473 (0.493; 2.4-4.0)	
	Medical secretary	3.407 (0.559; 2.4-4.0)		3.176 (0.601; 1.8-4.0)	
	Nursing assistant	3.188 (0.675; 1.0-4.0)		3.227 (0.345; 2.6-3.6)	
	Registered nurse	3.333 (0.466; 2.2-4.0)		3.363 (0.487; 2.0-4.0)	
**Staff eHLQ4: feel safe and in control**			2.953 (0.418; 1.8-4.0)		2.914 (0.418; 1.0-4.0)
	Medical physician	2.843 (0.436; 1.8-4.0)		2.838 (0.491; 1.0-3.8)	
	Medical secretary	3.069 (0.461; 2.2-4.0)		2.912 (0.451; 2.0-3.8)	
	Nursing assistant	2.987 (0.325; 2.2-3.6)		2.960 (0.275; 2.6-3.8)	
	Registered nurse	2.962 (0.407; 1.8-4.0)		2.934 (0.379; 2.0-4.0)	
**Staff eHLQ5: motivated to engage with digital**			2.783 (0.445; 1.6-3.8)		2.665 (0.439; 1.4-4.0)
	Medical physician	2.839 (0.482; 1.6-3.8)		2.675 (0.486; 1.4-4.0)	
	Medical secretary	2.821 (0.379; 2.0-3.6)		2.696 (0.487; 2.0-3.6)	
	Nursing assistant	2.880 (0.477; 1.8-3.8)		2.880 (0.413; 2.0-3.8)	
	Registered nurse	2.738 (0.446; 1.6-3.8)		2.604 (0.395; 1.6-4.0)	
**Staff eHLQ6: access to digital services that work**			2.566 (0.403; 1.5-3.8)		2.603 (0.411; 1.3-4.0)
	Medical physician	2.391 (0.461; 1.5-3.5)		2.417 (0.427; 1.3-3.3)	
	Medical secretary	2.661 (0.338; 2.2-3.7)		2.607 (0.333; 2.2-3.7)	
	Nursing assistant	2.778 (0.325; 2.0-3.3)		2.833 (0.383; 2.2-3.7)	
	Registered nurse	2.589 (0.381; 1.7-3.8)		2.642 (0.393; 1.3-4.0)	
**Staff eHLQ7: digital services that suit individual needs**			2.549 (0.508; 1.0-4.0)		2.510 (0.506; 1.3-4.0)
	Medical physician	2.321 (0.499; 1.0-3.5)		2.229 (0.489; 1.3-3.0)	
	Medical secretary	2.741 (0.381; 2.0-3.5)		2.470 (0.435; 2.0-3.3)	
	Nursing assistant	2.783 (0.352; 1.8-3.0)		2.800 (0.368; 2.0-3.3)	
	Registered nurse	2.572 (0.535; 1.0-4.0)		2.597 (0.498; 1.3-4.0)	

^a^eHLQ: eHealth Literacy Questionnaire.

### Before Implementation

Before implementation, the score of eHLQ2 (*understanding of health concepts and language*) showed significant differences among the 4 groups (*F*_3,185_=4.47; *P*=.005). The medical physicians scored significantly higher than the medical secretaries (Tukey test, *P*=.02) and nursing assistants (Tukey test, *P*=.01). There were no significant differences among the groups for eHLQ1 (*using technology to process health information*) and eHLQ3 (*ability to actively engage with digital services*).

The number of respondents who scored lower than two-thirds of the maximum score (2.67) varied between 22% (10/46) and 31% (5/16) among the staff groups for eHLQ1 (*using technology to process health information*), with medical physicians representing the lowest and nursing assistants the highest percentage. For eHLQ2 (*understanding of health concepts and language*), the percentage varied from 2% (1/46) to 17% (5/29) and in eHLQ3 (*the ability to actively engage with digital services*), from 6% (1/16) to 10% (3/29), with medical physicians representing the lowest percentage again but now with the medical secretaries representing the highest percentage <2.67 in both scales.

The scores of staff eHLQ4 (*feel safe and in control*) and staff eHLQ5 (*motivated to engage with digital services*), which relate to the perception of the use of the system, were lower than the scores in eHLQ1 to eHLQ3. There were no differences between the groups.

With regards to staff eHLQ6 (*access to digital services that work*) and staff eHLQ7 (*digital services that suit individual needs*), which both reflect an overall experience with digital health services, the scores were even lower. Before implementation, for staff eHLQ6 (*access to digital services that work*), there were significant differences between the groups (*F*_3,183_=5.16; *P*=.002). Medical physicians had a significantly lower score than medical secretaries (Tukey test, *P*=.02), nursing assistants (Tukey test, *P*=.006), and registered nurses (Tukey test, *P*=.03). The abovementioned findings do not appear to be associated with differences in age or sex among the groups, as the only association between age and eHLQ scores was a small negative correlation for eHLQ3 (*ability to actively engage with digital services*; *r*=–0.2158; *P*=.003) and between males and females for staff eHLQ5 (*motivated to engage with digital services*; mean 3.027, SD 0.477 vs mean 2.739, SD 0.426; *P*=.001; effect size 0.637).

### After Implementation

After implementation, the scores of eHLQ2 (*understanding of health concepts and language*) differed among the professional groups (*F*_3,191_=4.72; *P*=.003), where medical physicians had a significantly higher score than medical secretaries (Tukey test, *P*=.001).

In addition, for staff eHLQ6 (*access to digital services that work*), the ANOVA test showed significant differences among the groups (*F*_3,190_=5.61; *P*=.001), where the medical physicians had a lower score than the nursing assistants (Tukey test, *P*=.002) and the registered nurses (Tukey test, *P*=.007). This pattern was repeated for the postimplementation measurement of staff eHLQ7 (*digital services that suit individual needs*), with significant differences among the groups (*F*_3,190_=8.51; *P*<.001), where the medical physicians had a significantly lower score than the nursing assistants (Tukey test, *P*<.001) and the registered nurses (Tukey test, *P*<.001).

After implementation, there was a negative correlation between age and four of the seven eHLQ scales: eHLQ1, *using technology to process health information* (*r*=–0.193; *P*=.007); eHLQ2, *understanding of health concepts and language* (*r*=–0.147; *P*=.04); eHLQ3, *ability to actively engage with digital services* (*r*=–0.263; *P*<.001); and staff eHLQ4, *feel safe and in control* (*r*=–0.153; *P*=.04). There was a difference between males and females in the eHLQ2 (*understanding of health concepts and language*; mean 3.548, SD 0.527 vs mean 3.376, SD 0.413; *P*=.04; effect size 0.394) and staff eHLQ5 (*motivated to engage with digital services*; mean 2.834, SD 0.500 vs mean 2.627, SD 0.417; *P*=.01; effect size 0.478).

### Association Between Self-reported Skills and eHLQ

To support the content validity, we tested whether there were any associations between eHLQ1 to eHLQ3 and what the respondent believed a colleague would describe their IT skills by calculating Pearson *r*. For the measurements before implementation, there were moderate to strong correlations among the three eHLQ scales and the IT skills item: eHLQ1 (*using technology to process health information*; *r*=0.2176; *P*=.004), eHLQ2 (*understanding of health concepts and language*; *r*=0.2522; *P*<.001), and eHLQ3 (*ability to actively engage with digital services*; *r*=0.4471; *P*<.001).

For the postimplementation measurements, there were similar correlations among the three eHLQ scales and the IT skills item: eHLQ1 (*using technology to process health information*; *r*=0.1926; *P*=.008), eHLQ2 (*understanding of health concepts and language*; *r*=0.2244; *P*=.002), and eHLQ3 (*ability to actively engage with digital services*; *r*=0.4429; *P*<.001).

This may be associated with the age of the respondents, as we also found a negative correlation between age and these 3 scales, as well as for the IT skills scale, as reported previously.

### Perceived Ease of Use

The respondents scored the lowest on the stability of IT system items before implementation (Table 5). This item was the only one to increase after implementation of the new EHR system, whereas the 2 others did not change, which partly confirms hypothesis 2.1, that the new EHR system would increase the perceived ease of use. When comparing the groups of medical staff before implementation for the item regarding sharing of data to reduce doublet registration, there were significant differences among the groups (*F*_3,185_=5.24; *P*=.002). Here, medical physicians had a lower score than medical secretaries (Tukey test, *P*=.005) and nursing assistants (Tukey test, *P*=.04). In addition, the registered nurses had a lower score than the medical secretaries (Tukey test, *P*=.04).

When comparing the groups of medical staff after implementation, there was still an overall significant difference between the groups with respect to their experience of data being shared between IT systems to reduce doublet registration (*F*_3,191_=7.48; *P*<.001). Medical physicians had a significantly lower score than nursing assistants (Tukey test, *P*<.001) and registered nurses (Tukey test, *P*=.005).

**Table 5:** Experience of quick and easy access, sharing of data to reduce doublet registration, and stability of IT^a^ systems.

Ease of use and staff			Before implementation, mean (SD; range)		After implementation, mean (SD; range)
**Quick and easy access to information**			2.964 (0.704; 1.0-4.0)		2.959 (0.625; 1.0-4.0)
	Medical physician	2.935 (0.6799; 1.0-4.0)		2.816 (0.697; 1.0-4.0)	
	Medical secretary	3.069 (0.7527; 1.0-4.0)		2.96 (0.611; 1.0-4.0)	
	Nursing assistant	2.75 (0.5774; 1.0-4.0)		3.0667 (0.594; 1.0-4.0)	
	Registered nurse	3.011 (0.7219; 1.0-4.0)		3 (0.594; 1.0-4.0)	
**Sharing of data to reduce doublet registration**			2.333 (0.719; 1.0-4.0)		2.3897 (0.705; 1.0-4.0)
	Medical physician	2.130 (0.7486; 1.0-4.0)		2.061 (0.8516; 1.0-4.0)	
	Medical secretary	2.6896 (0.7123; 1.0-4.0)		2.4 (0.5774; 1.0-4.0)	
	Nursing assistant	2.6875 (0.602; 1.0-4.0)		2.9333 (0.4577; 1.0-4.0)	
	Registered nurse	2.284 (0.694; 1.0-4.0)		2.456 (0.6227; 1.0-4.0)	
**Stability of IT systems**			2.089 (0.707; 1.0-4.0)		2.359 (0.721; 1.0-4.0)
	Medical physician	1.826 (0.7088; 1.0-4.0)		2.245 (0.829; 1.0-4.0)	
	Medical secretary	2.207 (0.675; 1.0-4.0)		2.32 (0.557; 1.0-4.0)	
	Nursing assistant	2.25 (0.7746; 1.0-4.0)		2.467 (0.6399; 1.0-4.0)	
	Registered nurse	2.126 (0.6879; 1.0-4.0)		2.379 (0.7017; 1.0-4.0)	

^a^IT: information technology.

When looking at the associations between the items for perceived ease of use and staff eHLQ5-7, hypothesis 2.2 was confirmed, as there were highly significant correlations before and after implementation. For the preimplementation measurements, the values were as follows: for the item *ease of access*, staff eHLQ5 (*r*=0.2831; *P*<.001), staff eHLQ6 (*r*=0.4385; *P*<.001), and staff eHLQ7 (*r*=0.4164; *P*<.001); for the item *data is shared between systems to reduce doublet registration*, staff eHLQ5 (*r*=0.2055; *P*<.001), staff eHLQ6 (*r*=0.4418; *P*<.001), and staff eHLQ7 (*r*=0.4165; *P*<.001); and for the item *stability of IT systems*, staff eHLQ5 (*r*=0.1753; *P*=.02), staff eHLQ6 (*r*=0.5519; *P*<.001), and staff eHLQ7 (*r*=0.4381; *P*<.001).

For the postimplementation measurements, the values were as follows: for the item *ease of access*, staff eHLQ5 (*motivated to engage with digital services*; *r*=0.3298; *P*<.001), staff eHLQ6 (*r*=0.5237; *P*<.001), and staff eHLQ7 (*r*=0.4759; *P*<.001); for the item *data is shared between systems to reduce doublet registration*, staff eHLQ5 (*r=*0.2763; *P*<.001), staff eHLQ6 (*r*=0.5122; *P*<.001), and staff eHLQ7 (*r*=0.5267; *P*<.001); and for the item *stability of IT system*s, staff eHLQ5 (*r*=0.3402; *P*<.001), staff eHLQ6 (*r*=0.4939; *P*<.001), and staff eHLQ7 (*r*=0.3869; *P*<.001).

## Discussion

### Principal Findings

This is the first in-depth examination of medical staff’s eHL and perception of ease of use in the transition from a combination of eHealth systems into an integrated EHR. We found that despite a systematic training program, extensive support, and implementation of a coherent EHR, the medical staff’s eHL did not change, except for a small decline in motivation. This is of interest, as the stability of the system is perceived to increase, and the perceived ease of access and the system’s ability to share data remain unchanged after the implementation of the EHR.

### eHL Scales

Our first hypothesis was an expected increase in all 7 scales of the staff eHLQ based on an increase in knowledge and skills obtained in the implementation process and an increase in the positive experience of using the new system. However, we were unable to confirm this hypothesis.

With respect to eHLQ1 to eHLQ3, relating to personal knowledge and skills, all groups of medical staff had relatively high scores compared with 2 recent studies on medical outpatients and nursing students [19,20]. Regardless of this, only a limited number considered themselves to be experts. Interestingly, there was a positive association between the scores of the eHLQ1 to eHLQ3 scales and the scale regarding how the respondents thought their colleagues would score their user level. This information adds to the evidence for the content validity of the eHLQ1 to eHLQ3 scales. All 4 scales were negatively correlated with the age of respondents after implementation. Interestingly, the association with age was less pronounced before implementation, where only eHLQ3 (*ability to actively engage with digital services*) was associated with age, and Pearson *r* was lower than that after implementation for the correlation of age and how they thought their colleagues would score their user level. This may indicate that the older part of the respondents experienced less confidence in their self-reported skills as an effect of their experience during the implementation of the new EHR system.

The medical secretaries and nursing assistants scored lower than the medical physicians, which may be related to their prior training or educational background. Such an association between the level of training or educational background has not yet been observed in relation to eHL; however, further exploration is needed to better understand the possible needs of stratifying digital capacity building.

Before the investigation, we expected that the medical staff’s knowledge and skills would increase during the implementation period because of the training and expected higher use of the systems. We were not able to identify such changes as evaluated by the eHLQ scores on scales eHLQ1 to eHLQ3 or in self-reported IT skills described by a colleague. Interestingly, the only change in the staff eHLQ scales was a small decrease in staff eHLQ5 (*motivated to engage with digital services*), indicating that the new EHR system appeared to be less beneficial for users.

As our findings suggest that medical staff report sufficient levels of knowledge and skills but are challenged in relation to how health technology and services are perceived and experienced, we suggest that training should focus on their existing assumptions and prior experiences with the existing EHR.

The lower scores of staff eHLQ5 to eHLQ7 further suggest that the training should focus on how the implementation of the EHR will increase the security and safety of patients, ensure data integration, and support workflows, with data being available to those who need them, including the patients at any time.

Despite the medical physicians having the highest scores in two of the three scales that relate to personal knowledge and skills (eHLQ2 [*understanding of health concepts and language*] and eHLQ3 [*ability to actively engage with digital services*]), they had the lowest scores among the groups of medical staff in three of the four scales relating to their trust in how data are handled (staff eHLQ4, *feeling safe and in control*) and experience with the services (staff eHLQ6 [*access to digital services that work*] and staff eHLQ7 [*digital services that suit individual needs*]).

On the basis of the mean value of eHLQ1 to eHLQ3, our results would suggest that the medical staff’s knowledge and skills are not the main issues to be addressed when planning the introduction of a new system. However, when looking at the distribution of scores, it is evident that for eHLQ1 (*using technology to process health information*), 22% (10/46) of medical physicians and 31% (5/16) of nursing assistants scored <2.67. A similar pattern occurred in eHLQ2 (*understanding of health concepts and language*) and eHLQ3 *(ability to actively engage with digital services*), albeit at a lower percentage below the value of 2.67. These results underline the importance of identifying subgroups with low scores across groups of medical staff to address their particular needs in relation to knowledge and skills.

### Perceived Ease of Use

Our second hypothesis was that the perceived ease of use, measured by experienced ease of access, ease of data shared between systems to avoid doublet registration, and stability of the system, would increase after the implementation of the EHR system. We only found an increase in the experience of stability of the IT system with the implementation of the new EHR, which should contribute to a higher perception of ease of use. For nursing assistants and registered nurses, we also found an increase, although not significant, in their score of experience of data being shared between systems to avoid doublet registration, which may be explained by a certain degree of support of their workflows in relation to data. In contrast, medical physicians tended to disagree more than other staff groups with the statement that data were shared between the systems to avoid doublet registrations. This indicates that the system before implementation did not sufficiently support the workflows of medical physicians, and as the medical physicians’ degree of disagreement increased after implementation, the new EHR did not have any beneficial effects on their workflows.

Our findings only partly support our hypothesis that the experience relating to the performance of the IT environment would improve within the initial short period of implementation of the first installation of the EHR. The experiences of quick and easy access to relevant information or sharing the data to reduce double registration did not improve overall. As the EHR is provided by one vendor and is anticipated to provide a better experience of coherence and easy access, it is of interest that the medical staff did not experience such an improvement. The new system has many new functions that support quick and easy access. We cannot exclude the possibility that more training and support could have increased the medical staff’s capacity to use the system, thereby improving their experience of quick and easy access to information by using macros and tailored interfaces, which the EHR supports.

We also hypothesized that we would be able to identify an association between the staff eHLQ5 to eHLQ7 scales and 1 or more of the 3 items reporting on perceived ease of use: quick and easy access, data being shared between systems to avoid doublet registration, and stability of the IT systems. We found such an association between all 3 staff eHLQ scales and all 3 perceived ease of use items both before and after the implementation.

Confirmation of the hypothesized associations between the staff eHLQ5 to eHLQ7 and the 3 items reporting on perceived ease of use contributes to a better understanding of how eHL, as understood by the eHLF model and measured by staff eHLQ, may intertwine with dimensions from the technology acceptance theory (ie, perceived ease of use and usefulness). User experience is a product of the individual’s competence, usability of the user interface, and level of complexity and difficulty of tasks to be solved.

Staff eHLQ5 (*motivated to engage with digital services*) discloses perspectives on the use of health technology, which may relate to a sense of ease of use and usefulness. Staff eHLQ6 (*access to digital services that work*) reports on the experience of data being available whenever needed, and independent of where you are, the data are provided by digital systems that work together. Staff eHLQ7 (*digital services that suit individual needs*) reports on the users’ feeling that the digital services suit their needs. In combination, the staff eHLQ5 to eHLQ7 report on this product at a generic level; however, in our study, they were largely influenced by the context of the old EHR or the new EHR system, respectively. User experience, and thereby the likelihood of adoption [13], is also influenced by the overall perception of how easy and how useful a given technology or system is.

In addition to the respondents’ level of eHL, by using the 3 items directly reporting on various aspects of ease of use, we also obtained a more detailed insight into the respondents’ specific experience of both the old EHR and the new EHR system and how this relates to their general motivation and experience with health technology.

### Implications

Our findings emphasize the need for caution when planning implementations of EHR, as recommended by the literature in this area, such as the studies by McAlearney et al [27] and Boonstra et al [9].

All 4 groups of medical staff had relatively low scores on the staff eHLQ scales, which relates to digital services, and this was most pronounced among medical physicians. If these data had been available to the vendor and the health care organization responsible for the training of the staff, it might have helped them to better address the specific needs of the users; in this case, the medical physicians were characterized by having a high level of self-reported knowledge and skills in relation to data and digital services.

Our findings may also contribute to the understanding of why medical physicians are often resistant to the implementation of EHRs. As pointed out by Boonstra et al [9], this also indicates that an increase in staff capacity with respect to increased digital knowledge and skills may not automatically contribute to an increase in user experience. This is supported by the Monkman and Kushniruk [13] model of adoption, where it is proposed that adoption and a good user experience are both related to the users’ eHL and the usability of the systems, as well as the main principle of TAM, which is that the perceived ease of use is a significant factor in facilitating acceptance and adoption [28]. This signifies the importance of tailoring the new EHR’s interface and the introduction of system functions according to the specific needs and competences of the medical staff.

The finding that respondents’ perception of the ease of access, data sharing to reduce doublet registration, and the stability of the IT systems are influenced by the respondents’ overall level of eHL to a large degree suggests that the users’ perception of systems is closely linked to not only their competence but also to their general experiences with and confidence in using technology. This knowledge leads us to recommend the identification of staff members with low staff eHLQ scores to better address this particular group specifically and help them during the training to develop or increase self-confidence and self-efficacy in their work with digital health technology.

### Limitations

The version of the staff eHLQ used in this study is not the final version and may need further validation. We had to exclude one item from this version as it was not modified to suit the domain to which it belonged. The eHLQ [16] has been thoroughly validated in several languages and appears to be a robust, valid psychometric instrument. The modifications made in the staff eHLQ do not change the intentions or the significant words of the individual items, and the Cronbach α for the scales demonstrates internal consistency similar to data obtained with the eHLQ. Therefore, we are confident that our results are reliable despite the use of this early version of the staff eHLQ. We also think that the content validity of the staff eHLQ scales is supported by the fact that the staff eHLQ5 and staff eHLQ6 mirror the experience of usefulness of the systems, and eHLQ1 to eHLQ3 is associated with self-reported IT skills, whereas staff eHLQ5 to eHLQ7 is associated with the experience of data being shared in a way that reduces the double entry of data. Another limitation is the lack of administration of the survey after the training but before the implementation of the new EHR system, as we were not able to distinguish between the effect of training and the influence of experiences with the new system, which may affect, for example, the motivation. The reason for this design was a naive approach, where we expected the implementation of the EHR to be beneficial; therefore, we only wanted to focus on the synergy of the new system together with training.

Unfortunately, we were not able to follow up with this after 12 months in the involved department because of restructuring. Therefore, we may have missed effects that would only occur after a longer period of observation, such as 6 or 12 months [29]. We still hope to be able to perform a follow-up later. This is now of particular interest as the vendor in February 2019 has installed a major revision that also has increased interoperability with other national services.

### Perspective

The digital competence of the medical staff may vary among countries and regions and may therefore be addressed differently when a vendor or organization introduces a new EHR system. The staff eHLQ may be used to better understand the particular needs of medical staff groups, which should be addressed.

In addition, staff eHLQ6 may have an important role in settings where the EHR is not only used for documentation of hospital activities but also for primary care activities, and data are expected to be available for all actors at any time. However, this requires further investigation.

The association between the level of eHL and indicators of how the respondents perceive the performance of the system calls for further research on whether ≥1 of the staff eHLQ scales (ie, eHLQ5-7) can be used as predictors for users’ acceptance of technology in health care settings.

### Conclusions

The staff eHLQ may be a good candidate for monitoring the medical staff’s response to their training during the implementation of a new EHR system. It may also inform those responsible for the implementation whether the process is not going according to plan, with respect to the staff’s knowledge, skills, trust in security, motivation, and experience of a coherent system that suits the needs and supports the workflow and data availability.

Overall, this new insight in the presented case could have been helpful for the organization that led the implementation of the EHR and helped them to understand how the training should focus on how to (1) make use of the new functionality, (2) inform about the changes in workflow, and (3) make sense of the transition and thereby focus less on digital competence. It should be noted that the lower scores of staff eHLQ5 to eHLQ7, as found in all groups of medical staff, may also be because of problems with the functionality of the EHR as it was the first installation.

This calls for both the vendors in their design and the health care organizations in their procurement to pay more attention to these areas in the implementation process.

## Abbreviations

eHL: eHealth literacy
eHLF: eHealth Literacy Framework
eHLQ: eHealth Literacy Questionnaire
EHR: electronic health record
ISCED: International Standard Classification of Education
IT: information technology
RQ: research question
TAM: Technology Acceptance Model
UTAUT: Unified Theory of Acceptance and Use of Technology
